# Zoledronic Acid Preserves Bone Structure and Increases Survival but Does Not Limit Tumour Incidence in a Prostate Cancer Bone Metastasis Model

**DOI:** 10.1371/journal.pone.0019389

**Published:** 2011-05-16

**Authors:** Tzong-Tyng Hung, Jeffrey Chan, Pamela J. Russell, Carl A. Power

**Affiliations:** 1 Prince of Wales Clinical School, Prince of Wales Hospital, Randwick, New South Wales, Australia; 2 Centre for Vascular Research, University of New South Wales, Sydney, New South Wales, Australia; 3 Faculty of Medicine, University of New South Wales, Sydney, New South Wales, Australia; 4 Australian Prostate Cancer Research Centre- Queensland, Princess Alexandra Hospital, Institute of Health and Biomedical Innovation, Queensland University of Technology, Brisbane, Queensland, Australia; University of Pennsylvania, United States of America

## Abstract

**Background:**

The bisphosphonate, zoledronic acid (ZOL), can inhibit osteoclasts leading to decreased osteoclastogenesis and osteoclast activity in bone. Here, we used a mixed osteolytic/osteoblastic murine model of bone-metastatic prostate cancer, RM1(BM), to determine how inhibiting osteolysis with ZOL affects the ability of these cells to establish metastases in bone, the integrity of the tumour-bearing bones and the survival of the tumour-bearing mice.

**Methods:**

The model involves intracardiac injection for arterial dissemination of the RM1(BM) cells in C57BL/6 mice. ZOL treatment was given via subcutaneous injections on days 0, 4, 8 and 12, at 20 and 100 µg/kg doses. Bone integrity was assessed by micro-computed tomography and histology with comparison to untreated mice. The osteoclast and osteoblast activity was determined by measuring serum tartrate-resistant acid phosphatase 5b (TRAP 5b) and osteocalcin, respectively. Mice were euthanased according to predetermined criteria and survival was assessed using Kaplan Meier plots.

**Findings:**

Micro-CT and histological analysis showed that treatment of mice with ZOL from the day of intracardiac injection of RM1(BM) cells inhibited tumour-induced bone lysis, maintained bone volume and reduced the calcification of tumour-induced endochondral osteoid material. ZOL treatment also led to a decreased serum osteocalcin and TRAP 5b levels. Additionally, treated mice showed increased survival compared to vehicle treated controls. However, ZOL treatment did not inhibit the cells ability to metastasise to bone as the number of bone-metastases was similar in both treated and untreated mice.

**Conclusions:**

ZOL treatment provided significant benefits for maintaining the integrity of tumour-bearing bones and increased the survival of tumour bearing mice, though it did not prevent establishment of bone-metastases in this model. From the mechanistic view, these observations confirm that tumour-induced bone lysis is not a requirement for establishment of these bone tumours.

## Introduction

Some primary cancers have a high propensity to metastasise to bone but the reasons for this remain unclear, although it is generally accepted that growth of most cancers in bone is dependent on factors present in the bone micro-environment. Tumour cells can induce proliferation and activation of bone cells through release of growth factors and this increase in activity causes further stimulation of the cancer cells in what has been termed a “vicious cycle” [1]. Bone involvement is common in prostate and breast cancers and myeloma, and while breast cancer and myeloma bone-metastases are predominantly osteolytic, involving increased osteoclast activity leading to bone erosion, prostate cancer bone metastasis cases are heterogeneous, involving both bone lytic and sclerotic effects [2]. In osteolytic lesions, cancer cells can produce cytokines that promote osteoclast differentiation directly [3], [4] and indirectly through stimulation of bone marrow stromal cells to promote osteoclastogenesis [5]. Similarly, tumour cells can produce cytokines that promote osteoblast activity and induce bone growth [6] and osteoblasts can induce cancer development and progression [7], [8], [9]. The interactions between the tumour cells and bone cells dictate the bone metastatic phenotype.

Zoledronic acid (ZOL) and other bisphosphonates (BP) inhibit the metastasis and growth of bone-resident tumours through inhibiting osteoclast function. Thus ZOL inhibits the bone-destructive effects of the tumour and the resulting inhibition of bone turnover also slows the vicious cycle, resulting in a decrease in bone-tumour size and, in some cases, the number of bone lesions in various animal models of osteolytic bone cancer [10]. However, there have been contradictory reports of the effects of ZOL on osteoblastic bone tumours. While ZOL treatment does not inhibit intra-osseous growth of the osteoblastic cell line LAPC-9 [11], the growth of intra-tibial LuCaP 23.1 cells is inhibited by ZOL [12]. A subsequent study suggested that tumour-mediated osteolysis is not required for osteoblastic metastases [11].

Here we use a mixed osteolytic/osteoblastic syngeneic mouse model of prostate cancer metastasis to bone, the RM1 bone metastatic (BM) model [13] to assess the impact of ZOL treatment on the *establishment* of bone metastases, the integrity of tumour-bearing bones and the overall survival of mice.

RM1(BM) cells injected into the left ventricle of the heart result in multiple bone metastases with few soft tissues tumours. We demonstrate that inhibition of osteolysis with ZOL treatment does not influence incidence of bony tumours. Nonetheless, ZOL treatment did increase survival of mice in a manner that is dose dependent. Most importantly, ZOL treatment also decreased the lytic effects of the RM1(BM) tumours on bone as assessed by histology and micro-computed tomography (micro-CT) analysis, and appears to inhibit calcification of tumour-induced endochondral bone, but not osteoid formation. We did not determine if ZOL influenced the size or growth kinetics of tumours. The experiments presented here employed intra-cardiac injection, which does not cause bone damage directly and the resulting metastatic foci in these experiments are likely to involve relatively few cells. Thus the establishment of metastases from single cells or small clusters of cells, a condition more likely to reflect natural metastatic events, does not necessarily require initial lytic conditions.

## Results

**Figure 1:**
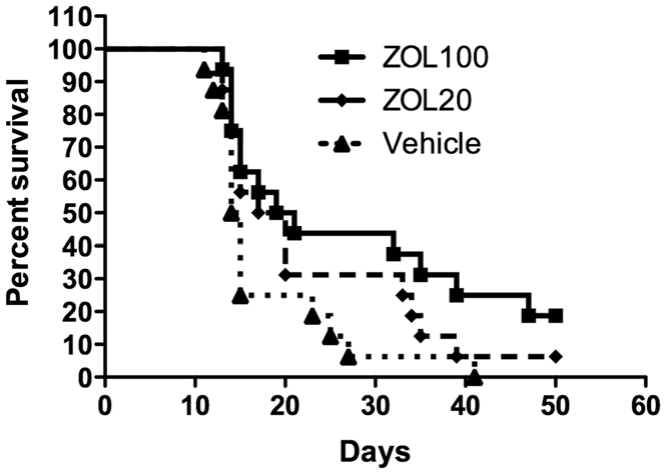
Zoledronic acid treatment improves mouse survival but does not reduce the number of metastases. (A) Kaplan-Meier survival plot of mice given an intra-arterial injection of RM1(BM) cells followed by treatment with the 100 µg/kg or 20 µg/kg zoledronic dosing regimens or vehicle only as described in Materials and Methods. The log rank test for trend indicates a trend of increased survival with increasing dose (p = 0.012), and a significant difference in survival between mice in the 100 µg/kg dose group vs vehicle control groups (p = 0.031, Kaplan-Meier followed by Breslow pair-wise comparison using SPSS 17.0).

In the RM1(BM) bone metastasis model, ZOL treatment had a significant effect on the survival of mice compared to untreated mice (Figure 1). Overall there was a trend of increased survival with increased dose (p = 0.012). Survival of mice in the 100 µg/kg dose was significantly different from that of the vehicle treated group (p = 0.031) while the 20 µg/kg dose did not reach a statistically significant difference from the control group with the sample sizes employed in these experiments (p = 0.108). The median survival time for the vehicle-, 20 µg/kg ZOL- and 100 µg/kg ZOL-treated mice was 14.5, 18.5 and 20 days respectively.

**Figure 2:**
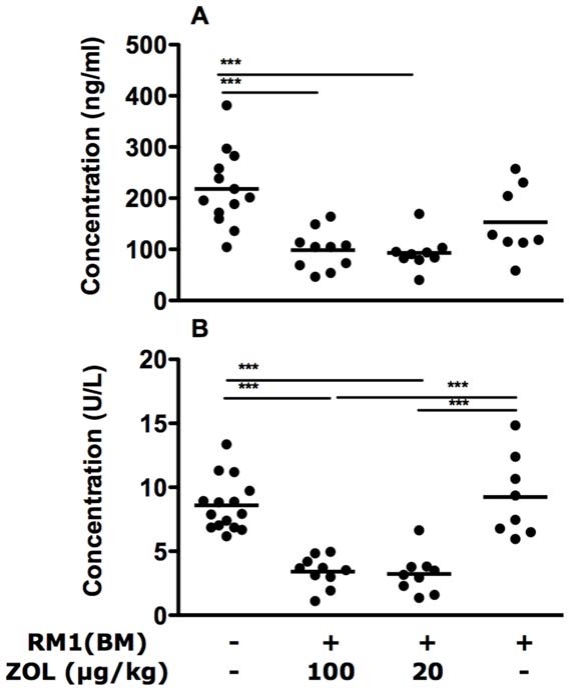
Surrogate markers of bone metabolism in serum of normal, tumor-bearing and zoledronic acid treated mice. (A) Levels of the osteoblast marker, osteocalcin were reduced in serum of zoledronic acid treated mice. (B*)* Tartrate-resistant acid phosphatase 5b, an indicator of osteoclast activity, was also reduced with treatment. Points on the graph represent serum levels for individual mice while bars show the position of the means within each group. Statistical analysis was performed using one-way ANOVA for analysis followed by Tukey's post test. *** p<0.001.

Osteocalcin levels were generally lower in tumour-bearing mice, though the difference was not statistically significant and treatment of these mice with 20 (p<0.01) or 100 µg/kg (p<0.01) of ZOL further reduced the serum osteocalcin compared to the normal controls (Figure 2A). The serum TRAP 5b levels of tumour-bearing, untreated mice were similar to those of the controls. However, treatment with 20 or 100 µg/kg of ZOL lowered the serum TRAP 5b to less than 40% of the control mice and those given the RM1(BM) cells only (p<0.001, Figure 2B).

**Figure 3:**
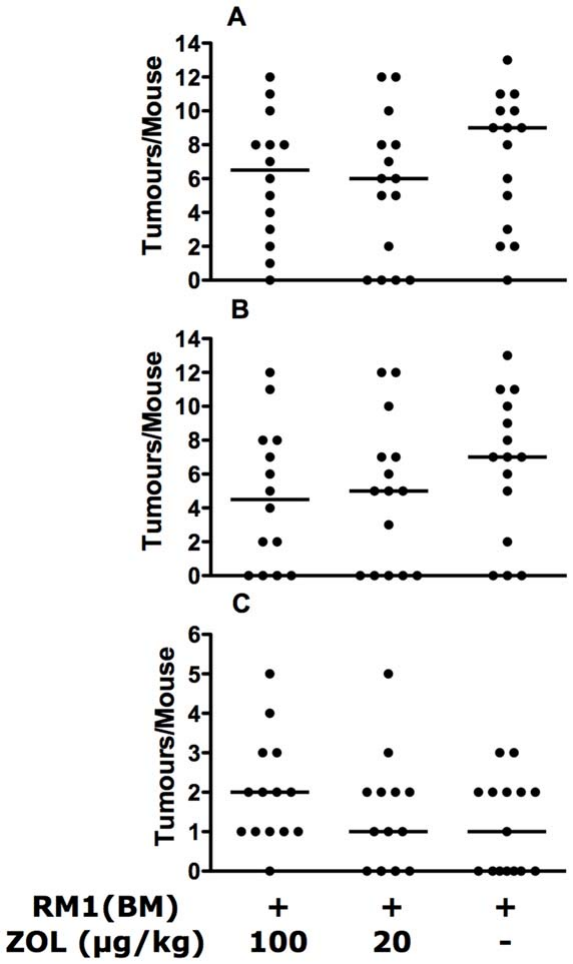
Zoledronic acid treatment did not reduce establishment of metastases in RM1(BM) injected mice. (A) Total number of metastases, (B) bone-metastases, or (C) soft tissue metastases as assessed by one-way ANOVA. Each point represents the number of metastases in an individual mouse, bars indicate the median within the group.

The presence of tumour lesions in mice was evaluated by two means, fluorescence imaging for green fluorescent protein (GFP) expression by tumour cells followed by confirmation with histology as described [13]. The RM1(BM) cell line was sensitive to ZOL treatment *in vitro* (Data not shown), but the treatment had little effect on the incidence of soft-tissue or bone tumours in this model. ZOL treatment did not reduce the number of bony lesions nor the number of tumour-metastases in soft tissue in the RM1(BM) injected mice (Figure 3A–C). We did not assess the effect of the ZOL treatment on the growth of established tumours.

**Figure 4:**
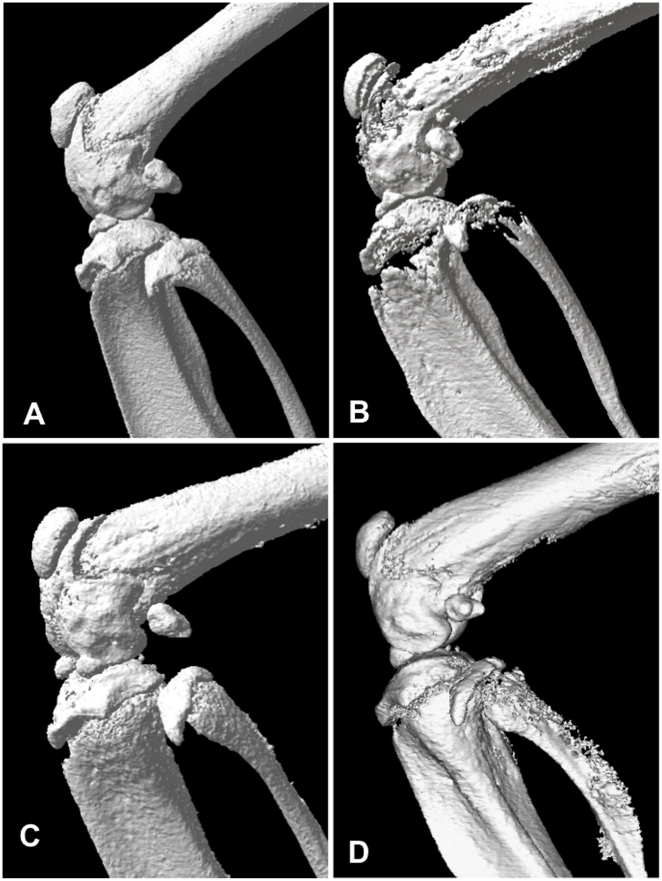
Zoledronic acid preserves bone structure. MicroCT scans of mouse femur and tibia; normal control (A), RM1(BM) tumour-bearing (B), RM1(BM) tumour-bearing treated with the 20 µg/kg ZOL regimen (C), RM1(BM) tumour-bearing treated with the 100 µg/kg ZOL regimen (D).

**Figure 5:**
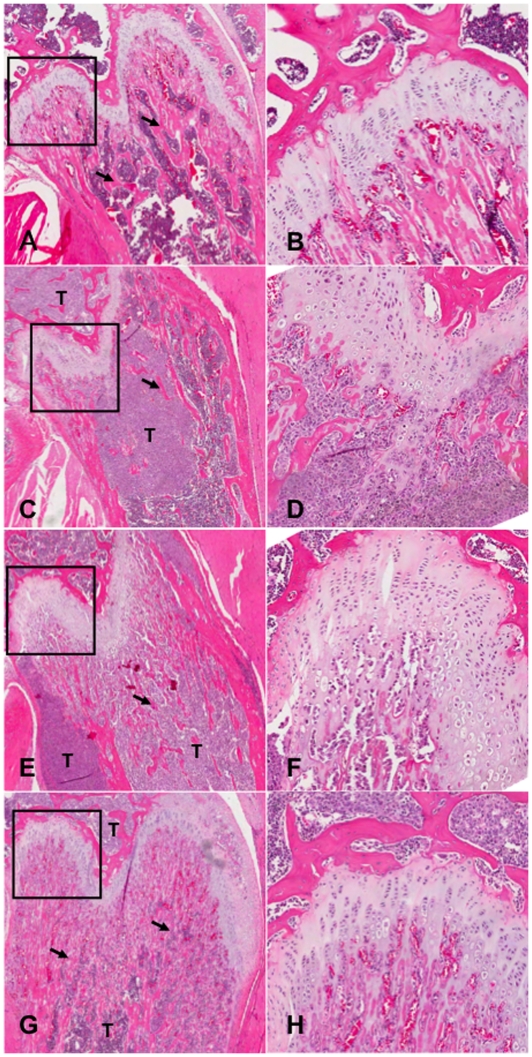
Histological analysis of the effects of zoledronic acid-treatment on normal and tumour-bearing bones. H&E stained sections of the femoral head at 40X (A, C, E and G) and 120X (B, D, F and H) magnification showing normal (A&B) and RM1(BM) tumour bearing bones (C–H); Typical examples of bones from untreated mice (A–D) and mice treated with 20 µg/kg ZOL (E&F) or 100 µg/kg ZOL (G&H) are shown. T = tumour, Arrow = Trabecular bone.

**Figure 6:**
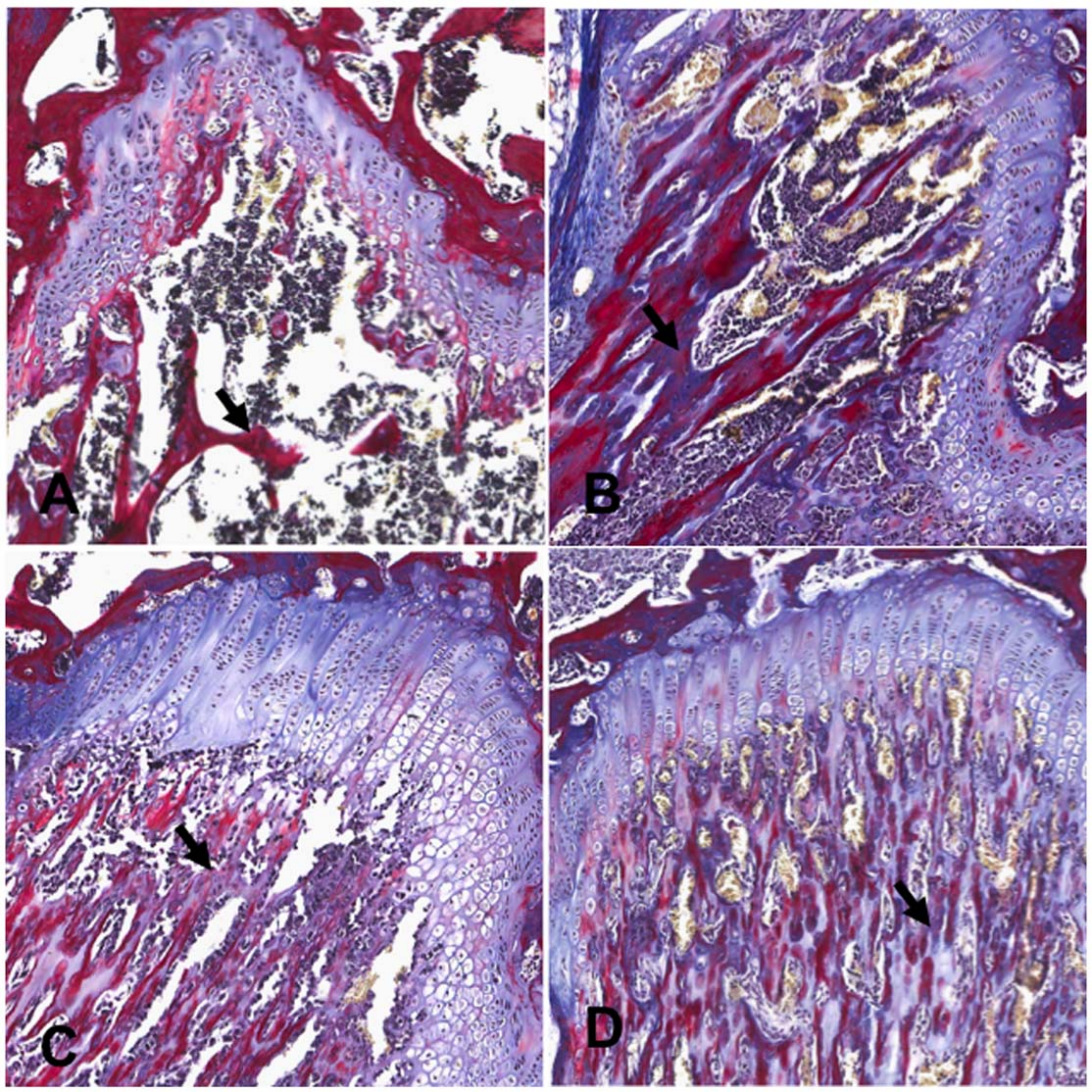
Histological analysis with tetrachrome stain for effects of zoledronic acid-treatment on normal and tumour-bearing bones. Tetrachrome stain for differentiation of mineralised and unmineralised bone at 120x magnification Normal (A), or RM1(BM) tumour bearing bones (B–D). (A&B) Untreated, (C) 20 µg/kg ZOL (D) 100 µg/kg ZOL. T = tumour, Arrow = Trabecular bone.

Reconstructed three-dimensional images of bones from micro-CT and representative H and E stained histological sections are shown in Figure 4 and 5 respectively. The sections were also stained with tetrachrome stain [14] to distinguish new bone development from established/calcified bone (Figure 6). Mice with metastatic bone lesions in the long bones showed significant bone lysis, particularly near the epiphyseal plate, with a reduction in trabecular bone (Figure 4B) compared to the normal controls (Figure 4A). The lytic effect of the tumour is reduced in the 20 µg/kg ZOL treatment group (Figure 4C) and perhaps more so in mice given 100 µg/kg ZOL (Figure 4D). The micro-CT results are confirmed by the histological results (Figure 5 and 6), showing normal trabecular bone structure in the untreated control mice (Figure 5A, 5B and 6A) but evidence of both lytic and sclerotic effects in the untreated tumour-bearing bones (Figure 5C, 5D and 6B). ZOL treatment at 20 µg/kg preserved trabecular structure in tumour-bearing bones (Figure 5E, 5F and 6C) and the 100 µg/kg dose increased trabecular bone (Figure 5G, 5H and 6D). The increase in tumour-induced endochondral trabecular bone formation is particularly evident in the ZOL treated mice (Figure 5E and 5G) due to the concomitant inhibition of tumour-induced lysis. However, incomplete mineralization of trabecular bone osteoid near the epiphyseal plate is evident in the ZOL-treated tumour-bearing bones (Figure 6C and 6D).

**Figure 7:**
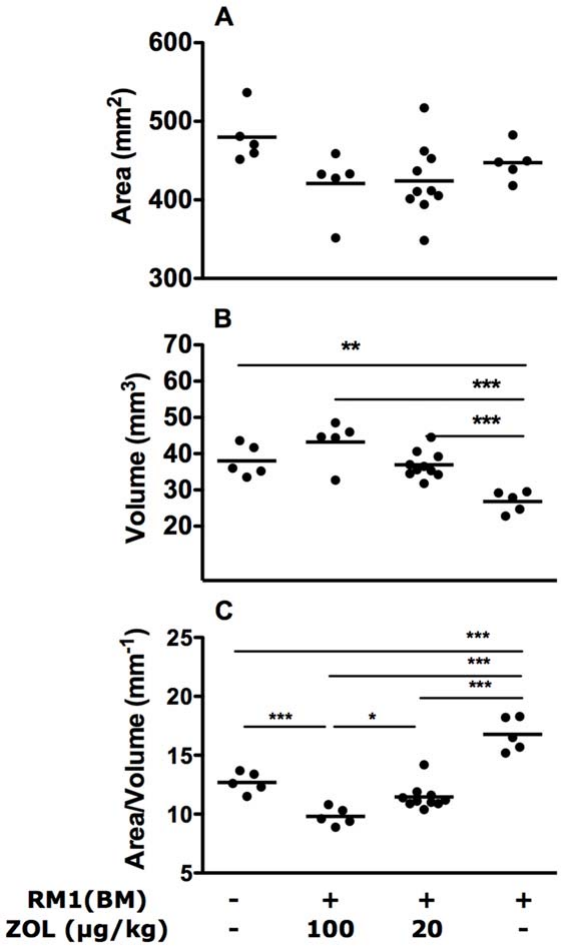
Zoledronic acid treatment alters bone volume and the bone surface-area:volume ratio in normal and tumour-bearing bones. There was no significant change in bone surface area (A) induced by the presence of a tumour or treatment with zoledronic acid. However, bone volume was dramatically reduced in RM1(BM) containing bones and the treatment with ZOL prevented this loss in a dose-dependent manner (B), resulting in lower surface area/volume ratio (increased bone density) compared to normal or tumor-bearing bones (C). Results are based on measurements obtained from CT scans as described in Materials and Methods section. Statistical analysis was performed by one-way ANOVA followed by Tukey's post test, * p<0.05, ** p<0.01, *** p<0.001.

Quantitative analysis of micro-CT data indicates that there was no significant change in the surface area of the bones in normal, tumour-bearing or treated bones (Figure 7A). The primary effect of the tumour is the reduction in bone volume (p<0.01, Figure 7B) and consequent increase in the bone-surface area: bone-volume ratio in tumour-bearing bones (p<0.001, Figure 7C), while the ZOL treatments result in an increase in bone volumes (p<0.001, Figure 7B) and a decrease in bone-surface area: bone-volume ratio (p<0.001, Figure 7C) of tumour-bearing bones.

## Discussion

When injected by the intra-cardiac route, the RM1(BM) cell line induces multiple bone metastases with relatively few (and relatively small) soft tissue tumours [13]. Of note is the observation that the resulting bony metastases do not show preference for particular skeletal sites where high bone turnover occurs, indicating that high bone turnover is not required for the establishment of metastases. Furthermore the resulting bone metastases have mixed osteolytic and osteoblastic characters with neither phenotype predominating [13]. We determined that intra-cardiac injection of RM1(BM) cells is an excellent model for assessing if ZOL could inhibit the establishment of tumours metastases which were not dependent upon lytic growth, that is, the vicious cycle. We also assessed the ability of ZOL treatment to inhibit the tumour-induced osteolysis and endochondral bone formation and determined the influence of the treatment on the overall survival of mice.

Like other nitrogen-containing BPs, ZOL induces apoptosis in osteoclasts by inhibiting enzymes of the mevalonate pathway and preventing the isoprenylation of small GTP-binding proteins such as Ras and Rho [15], [16]. *In vivo*, BPs bind with high affinity to bone hydroxy-appatite and thus are sequestered by active osteoclasts in high concentrations, leading to osteoclast apoptosis. Although a number of other mechanisms including direct killing of cancer cells, inhibition of angiogenesis and activation of γδT cells [17], [18] have been demonstrated for ZOL, its effect on osteoclasts remains the main mechanism by which ZOL inhibits bone cancer under current treatment regimens. The benefit to the patient is potentially two-fold: ZOL inhibits the osteoclast-mediated lysis induced by the tumour and so interrupts the vicious cycle and subsequent promotion of tumour growth. This is certainly true of osteolytic tumours, but the effects of ZOL treatment on tumours that are not dependent upon bone lysis are poorly understood.

Treatment of tumour-bearing mice with ZOL prolonged the survival of mice in our study (Figure 1). At the 100 µg/kg dose, there is a statistically significant increase in survival time compared to the vehicle treated mice and a trend towards increased survival in the 20 µg/kg treated mice. Similar results have been reported for other syngeneic cancer models [7], [8], [9]. However in osteolytic models of multiple myeloma [10] and breast cancer [8], significant reduction in establishment of bone metastatic lesions was observed, but this is not true for our system (Figure 3). We have demonstrated previously [13] that the RM1(BM) cells metastasise to most skeletal sites and thus metastasis of these cells to bony sites appears to be independent of bone turnover rates. Thus, we are not surprised that the inhibition of bone lysis by ZOL treatment has no demonstrable effect on the establishment of tumours in bone. These findings suggest that the predilection of the RM1(BM) cells for bone is dependent upon other factors, potentially an increased homing to bony sites or dependence upon factors independent of bone lysis. Although this is a mixed osteolytic/osteoblastic model, the results are in contrast to those of Corey [12], but similar to results achieved with the osteoblastic cells line, LAPC-9 [11].

We have assessed the concentrations of the two bone turnover markers, osteocalcin (secreted by osteoblasts) and TRAP 5b (produced by osteoclasts) in serum of experimental mice. ZOL treatment decreases the serum levels of both these markers (Figure 2) indicating that ZOL has inhibited both osteoclast and osteoblast activity at the systemic level. These results are in agreement with previous reports of decreased osteocalcin in breast cancer patients [19] and a TRAP 5b decrease in mice [20] following treatment with ZOL. The finding that both osteoblast and osteoclast activity are impaired suggests that the observed increase in bone volume and surface area with ZOL treatment (Figure 7) is a result of reduced osteoclast activity, rather than increased osteoblast activity. However, we find it particularly interesting that the osteocalcin levels in serum are decreased in ZOL treated tumour-bearing mice attendant with the observation of increased endochondral osteoid formation in these mice (Figure 5 F and H). Importantly, the new osteoid material observed in the ZOL treated mice appears largely un-calcified (Figure 6C and 6D), particularly at the high dose (Figure 6D), at the time when the mice were killed suggesting an uncoupling of the osteoid development and calcification steps of bone formation. Of relevance is the observation that osteocalcin-deficient mice have increased bone formation [21]. The decrease in trabecular bone calcification with increased in ZOL dose is a novel finding and appears to contradict the extensive existing literature on the effects of bisphosphonates on bone. However, we also show that ZOL treatment of tumour bearing mice increases the bone volume of mouse leg bones (Figure 7B), which supports previous findings. In similar preclinical studies using the C4-2 [21] and LuCaP 23.1 [22] xenograft models, ZOL treatment was also shown to increase the overall bone volume. Thus, the decreased mineralization of newly formed trabecular bone observed here is consistent with an overall preservation of existing mineralized bone in tumour bearing mice. These effects of ZOL in maintaining bone integrity are associated with increased survival in ZOL treated mice.

The effects of ZOL on the bone structure may or may not directly affect the progression of the cancer, but prevention of abnormal bone modeling in a tumour-ridden bone may be key to ZOL reducing the risk of skeletal related complications and prolonging the time to skeletal related event occurrence in patients with bone-metastatic prostate cancer [23], [24]. Furthermore, ZOL treatment has significant implications for dealing with pain reduction in the clinic. BPs improve the bone structure in patients, with a major reduction in related pain [25], [26], [27], which has been attributed to the re-calcification of the bone, that has led to a decrease in analgesic usage. Of relevance is the observation in multiple myeloma (an osteolytic disease) patients, decreased osteocalcin levels, as reported here, was related to reduction in skeletal related events [28]. Treatment with zoledronic acid is an effective therapy for mixed osteoblastic/lytic tumours as it increases survival of treated mice. The treatment inhibits bone lysis and reduced osteoclast activity as expected. ZOL treatment also reduced serum levels of osteocalcin, but did not inhibit tumour-induced endochondral osteoid formation although it partially inhibited subsequent calcification of this osteoid material in a dose-dependent manner. ZOL did not reduce the frequency of metastases indicating that although RM1(BM) tumours induce bone lysis, establishment of tumours is not dependent upon lytic growth.

## Materials and Methods

### Ethics statement

The University of New South Wales Animal Care and Ethics Committee approved animal housing, husbandry, and all experimental procedures performed on animals prior to experiments (Project application approval number: 05/120B). Mice were monitored closely (on a daily basis) and euthanased according to predefined criteria (loss of 20% of body weight, significant loss of condition, or partial paralysis) as approved by the animal ethics.

### Cell culture

RM1 cells were obtained from Dr. T Thompson, Baylor College, and RM1(BM) was developed by serial passaging of RM1 *in vivo* [13]. Both cell lines were cultured under standard conditions (37°C in a 5% CO_2_ incubator) in DMEM (Invitrogen, Melbourne, Australia) with 10% FBS (Invitrogen) and L-glutamine (Invitrogen).

### Surrogate Markers of Bone Metastasis in Serum

Sera from tumour-cell injected mice and normal control mice collected at necropsy by cardiac puncture were assessed for the presence of surrogate markers of bone metabolism. Insufficient serum was available from some mice for assessing surrogate markers. Osteocalcin content was assessed using an osteocalcin enzyme-linked immunosorbent assay (ELISA) (Biomedical Technologies Inc., Stoughton, MA) according to the manufacturer's instructions. Tartrate-resistant acid phosphatase (TRAP) 5b concentration was determined by the MouseTRAP assay (Suomen Bioanalytikka Oy, Turku, Finland) as proscribed in the kit. Serum protein levels were determined by comparison to a standard curve prepared from standards provided with the assays.

### Animals

5–6 week old C57BL/6 mice were obtained from the Animal Resource Centre (Adelaide, Australia). They were housed and maintained at the Biological Resources Centre, Little Bay, NSW. On day 0 of the experiment, mice were injected with either of 3×10^4^ RM1(BM) cells in saline or saline alone (100 µl) into the left ventricle of the heart. Mice were treated on days 0, 4, 8 and 12 via subcutaneous injection of either 20 µg/kg or 100 µg/kg of zoledronic acid (Novartis Pharmaceuticals, Australia), or saline (vehicle). The cumulative dose at 20 µg/kg equates to an approximate single dose of 4 mg in patients. Mice were monitored closely and euthanased according to predefined criteria (loss of 20% of body weight, significant loss of condition, or partial paralysis) as approved by the animal ethics. The number of tumours in each mouse was assessed by GFP fluorescence (as described previously [13]) at necropsy followed by histology. The presence of only a thoracic tumour is an indication that the IC injection was not successful and therefore such mice were excluded in these assessments.

### Histology

The mouse carcasses were fixed in 10% formalin in phosphate-buffered saline (PBS) for 48 h then decalcified in 10% EDTA for 10–14 days. Decalcified fore and hind limbs were embedded in paraffin after tissue processing (dehydration, clearance and impregnation). Sections (5 µm) were cut from each block and stained with Harris' Hematoxylin and Eosin (H&E). For analysis of mineralization of the bone, sections (8 µm) were cut and stained with tetrachrome stain according to previously published methods [14] with slight modifications: the ponceau red solution was replaced with Briebrich Scarlet/Acid Fuchsin solution. Sections were examined with the Aperio Scanscope XT (CA, USA) and images taken with Imagescope Viewer (Aperio, CA, USA).

### Micro-CT scanning and analysis

After formalin fixation, the hind limbs were selected for micro-CT scanning before processing for histology. Tumour-bearing limbs were identified prior by expression of GFP *in vivo*. For tumour-bearing mice receiving the 20 µg/kg dose regimen, both right and left hind legs of five mice were scanned. For other treatment groups, only a single leg was scanned from each mouse. All analysed bones were from animals that were culled between day 15 and 21 after injection of the cells. Scanning and reconstruction was performed using a Skyscan 1072 micro-CT system and associated software (Skyscan, Antwerp, Belgium). While maintaining the specimen in the field of view during the image acquisition, the sample was rotated 180° around the vertical axis. Images were exposed for 5.9 sec and projections recorded at each rotational step, every 0.9°, at 80 keV and 100 mA. Cross sectional images (DICOM format) were generated from the projected CT images using Nrecon (Skyscan, Belgium). Bone surface area and volume were calculated from DICOM images using the manufacturer's software (CT-Analyzer, Skyscan, Belgium). The region of the leg around the knee joint from the apex of the third trochanter of the femur to the bifurcation of the tibia and fibula was selected for this analysis to ensure consistency. Reconstruction of the 3D model and image visualisation was performed using ANT software (Skyscan, Belgium).

### Statistical analysis

One-way ANOVA followed by Tukey's post tests were performed using GraphPad Prism version 4.00 for MacIntosh, GraphPad Software (San Diego, USA). Survival was assessed by Kaplan-Meier followed by Breslow pair-wise comparison using SPSS 17.0.
